# Blood pressure trajectories during pregnancy and associations with adverse birth outcomes among HIV-infected and HIV-uninfected women in South Africa: a group-based trajectory modelling approach

**DOI:** 10.1186/s12884-020-03411-y

**Published:** 2020-11-30

**Authors:** Thokozile R. Malaba, Annibale Cois, Hlengiwe P. Madlala, Mushi Matjila, Landon Myer, Marie-Louise Newell, for the PIMS Study Group

**Affiliations:** 1 Division of Epidemiology and Biostatistics, School of Public Health and Family Medicine, University of Cape Town, Cape Town, South Africa; 2 Burden of Disease Research Unit, South African Medical Research Council, Cape Town, South Africa; 3 Division of Maternal Fetal Medicine, Department of Obstetrics and Gynaecology, University of Cape Town, Cape Town, South Africa; 4 Centre for Infectious Disease Epidemiology and Research, School of Public Health and Family Medicine, University of Cape Town, Cape Town, South Africa; 5 School of Human Development and Health, University of Southampton, Southampton, UK; 6 School of Public Health, University of the Witwatersrand, Johannesburg, South Africa

**Keywords:** Blood pressure levels, Pregnancy, Preterm delivery, Low birthweight, Group-based trajectory modelling

## Abstract

**Background:**

High blood pressure (BP) late in pregnancy is associated with preterm delivery (PTD); BP has also been associated with HIV and antiretroviral therapy (ART), but whether the relationship between BP assessed longitudinally over pregnancy and PTD and low birthweight (LBW) is modified by HIV/ART is unclear. We hypothesise the presence of distinctive BP trajectories and their association with adverse birth outcomes may be mediated by HIV/ART status.

**Methods:**

We recruited pregnant women at a large primary care facility in Cape Town. BP was measured throughout pregnancy using automated monitors. Group-based trajectory modelling in women with ≥3 BP measurements identified distinct joint systolic and diastolic BP trajectory groups. Multinomial regression assessed BP trajectory group associations with HIV/ART status, and Poisson regression with robust error variance was used to assess risk of PTD and LBW.

**Results:**

Of the 1583 women in this analysis, 37% were HIV-infected. Seven joint trajectory group combinations were identified, which were categorised as normal (50%), low normal (25%), high normal (20%), and abnormal (5%). A higher proportion of women in the low normal group were HIV-infected than HIV-uninfected (28% vs. 23%), however differences were not statistically significant (RR 1.27, 95% CI 0.98–1.63, reference category: normal). In multivariable analyses, low normal trajectory (aRR0.59, 0.41–0.85) was associated with decreased risk of PTD, while high normal (aRR1.48, 1.12–1.95) and abnormal trajectories (aRR3.18, 2.32–4.37) were associated with increased risk of PTD, and abnormal with increased risk of LBW (RR2.81, 1.90–4.15).

**Conclusions:**

While HIV/ART did not appear to mediate the BP trajectories and adverse birth outcomes association, they did provide more detailed insights into the relationship between BP, PTD and LBW for HIV-infected and uninfected women.

**Supplementary Information:**

The online version contains supplementary material available at 10.1186/s12884-020-03411-y.

## Background

Highly elevated blood pressure (BP) levels during pregnancy affect 10–15% of all pregnancies [1, 2]. Hypertensive disorders in pregnancy (HDP), including gestational and chronic hypertension, pre-eclampsia and eclampsia, are associated with increased risk of maternal and neonatal morbidity and mortality [1, 3]; specifically increased risk of adverse pregnancy outcomes including fetoplacental insufficiency, preterm delivery (PTD) and low birth weight (LBW) infants [4, 5]. Differential HDP risk has been theorised to exist in immunocompromised states, such as HIV-infection in pregnancy especially with antiretroviral therapy (ART) use. This was demonstrated in a recent systematic review showing increased HDP risk in HIV-infected women on ART, particularly those using protease inhibitors (PI) based regimens, compared to untreated HIV-infected women [6]. In addition to concerns around increased HDP risk, both untreated advanced HIV infection [7, 8] and ART use [9–11] have also been associated with increased risk of adverse pregnancy outcomes.

Accurate blood pressure measurement is vital to identify HDP, guiding diagnosis, antihypertensive treatment and timing of delivery. Clinically, BP is routinely measured throughout the antenatal period [12], with the expectation that BP will decrease steadily until mid-pregnancy and then increase slightly again until delivery [13]. However, in population-based research studies BP is typically analysed using discrete, cross-sectional assessments at arbitrary time points (such as at the first antenatal care (ANC) or enrolment visit) [14], with high BP defined by cut-off points used in non-pregnant adults. Characterising BP trajectories during pregnancy in population-based studies may better inform understanding of the mechanisms driving BP changes in pregnancy and development of HDP, and their timing, than cross-sectional assessments. BP trajectories may also provide more accurate insights into the relationship between BP and maternal, perinatal and neonatal outcomes than cross-sectional assessments and could thus inform development of interventions for improvement in HDP management.

Elucidating the HIV/ART and HDP relationship is critical to understanding the etiology of HDP; however, studies investigating this association have generally used traditional hypertension cut-offs from BP readings at first ANC visit. Contrary to the use of serial BP measurements in clinical care, few studies have assessed BP trajectories across pregnancy to identify women at risk of developing HDP and/or adverse pregnancy outcomes. We hypothesised that within a cohort of pregnant women there are groups of individuals that follow distinctive BP trajectories that may not be identifiable using traditional cross-sectional hypertension cut-offs; and that these may differ according to HIV/ART status. The aim of this analysis was to describe the natural history of blood pressure throughout pregnancy in HIV-infected and HIV-uninfected women and assess whether this mediates the association between ART use and adverse pregnancy outcomes.

## Methods

### Study population

Between April 2015 and October 2016, consecutive pregnant women (aged ≥18 years), regardless of their HIV status, making their first ANC visit at a large public sector primary care facility in Cape Town, South Africa were enrolled in a prospective cohort [15].

As part of routine ANC services all women had systolic (SBP) and diastolic (DBP) blood pressure measured using automated monitors (Edan M3A Vital Signs Monitor), at the first and subsequent ANC visits. For BP, there was a minimum of 5 min rest before a single measurement took place and women were seated upright with the arm supported and the use of appropriately sized cuffs for arm circumference (25–35 cm and 35.5–46 cm cuffs used). In line with national guidelines for primary health care settings, as part of routine care women diagnosed as hypertensive without risk factors, proteinuria or symptoms were started on anti-hypertensives (alpha-methyldopa), while, those with risk factors (as per ISSPH guidance [16]) but no proteinuria were started on low-dose aspirin [17]. Hypertensive women with proteinuria were referred to nearest secondary or tertiary hospital and started on magnesium sulphate if deemed necessary [17].

BP and GA were measured by regularly trained ANC midwives, and recorded in the clinical records. Gestational age (GA) was routinely established at the first ANC visit based on last menstrual period (LMP) and symphysis-fundal height (SFH), with any woman clinically assessed (LMP and/or SFH) to be ≤24 weeks gestation referred for a same day ultrasound by the PIMS research sonographer using standardized assessment protocols and blinded to the midwife GA assessment.

HIV-infected women all on ART either initiated ART pre-conception, continuing their current regimen throughout pregnancy, or initiated ART during pregnancy; with the predominant regimen being a fixed-dose combination of TDF + FTC + EFV.

### Data collection

Following the first ANC visit, data from enrolled women were abstracted from clinical records including HIV status, pregnancy history (previous and index pregnancies), medications prescribed, and any maternal diagnoses during pregnancy. Following delivery, all BP recordings during pregnancy (including measurement date) and obstetric outcomes (including date of delivery and birthweight), were abstracted from clinical records. To avoid inclusion of spuriously high values, all measurements occurring during labour in inpatient settings were excluded because BP medications may be temporarily withheld or because labour-related stress or other emergent conditions may cause temporary elevations in BP. Because of repeated BP measurements during acute and unstable periods throughout pregnancy, 13 GA time point windows were set up with intervals corresponding with ANC visit intervals (4 week intervals until 32 weeks GA after which 2 week intervals until 41 completed weeks). Within each of the 13 GA time point windows, the BP measurement was allocated based on the first available measurement, discarding any remainder in that window.

### Outcomes

Prehypertension was defined as BP readings with SBP from 120 to 139 mmHg and/or DBP from 80 to 89 mmHg; hypertension was defined as SBP ≥140 mmHg and/or diastolic pressure ≥ 90 mmHg on more than two occasions [18], and classified according to gestational age at onset. Hypertension classification was according to ISSHP guidelines [16], but based only on BP measurements. We were unable to take proteinuria into account as part of our diagnostic criteria because screening results (urine dipstick) are not systematically recorded in routine clinical records, similarly other biomarker results were not available. BP was classified as chronic if assessed < 20 weeks GA (assuming this existed pre-conception); and as gestational when initially normotensive at < 20 weeks and then hypertensive > 20 weeks or assessed for the first time > 20 weeks hypertension. Non-severe hypertension was defined as SBP from 140 to 159 mmHg and/or DBP from 90 to 109 mmHg) and severe hypertension defined as SBP ≥160 and/or DBP ≥110 mmHg). Mean arterial pressure (MAP) was calculated using the formula: MAP = (SBP + 2 x DBP)/3 [19].

Secondary outcomes included PTD and LBW. PTD was defined as delivery at < 37 weeks’ gestation, categorized as late preterm (34–37 weeks), moderately preterm (32–34 weeks) or very preterm (< 32 weeks), as assessed by the most reliable available GA assessment method at baseline (ultrasound, SFH or LMP). LBW was defined as birthweight < 2500 g and very low birth weight (VLBW) as < 1500 g. Pregnancies not resulting in the delivery of a live born infant were defined as a pregnancy loss.

### Statistical analyses

Sample characteristics are described as median and interquartile range for numerical variables and frequency for categorical measures. Group-based trajectory modelling (GBTM), was used to analyse changes in participants’ SBP and DBP during pregnancy [20]. GBTM is an application of finite mixture modelling, allowing the identification of population subgroups (classes) characterised by statistically distinct trajectories for one or more outcomes of interest. In each class, the outcomes trajectories are summarised by polynomial functions of time [21]. Model parameters are calculated by maximising a likelihood function, and include estimates of individual probabilities of class membership and of the polynomial coefficients which define the shape of the trajectories for each class [22]. In addition to estimates of class membership probability, the joint model also estimates conditional probability linking membership across the trajectory classes of both outcomes of interest [22]. We applied GBTM to identify classes of pregnant women characterised by different joint trajectories for SBP and DBP during pregnancy. We chose the optimal number of classes and the degree of the polynomials used to represent the trajectories by fitting multiple models with different combinations of these parameters, and comparing their fit to the data through the Bayesian information criterion (BIC), which balances model fit and complexity, with smaller BIC values indicating a better fit [21]. Linear, quadratic and cubic polynomial were tested, and number of classes varying between 1 and 7.

After fitting the chosen model, women were assigned to SBP and DBP classes based on the highest-class membership probability. The probability matrix of joint class membership and considerations of clinical significance of the observed differences were then used to assign women to ‘joint groups’ (groups hereafter) characterised by different combinations of SBP and DBP trajectories, with descriptive labels assigned based on the visual patterns of BP changes throughout pregnancy. Multinomial regression was then used to identify predictors of group membership, and Poisson regression with robust error variance [23] to investigate the association between the group membership and adverse birth outcomes (PTD and LBW).

Statistical analyses were performed using STATA version 14.0 (Stata Corporation, College Station, TX, USA) and its TRAJ plugin to perform GBTM [22]. Two-tailed chi-squared and rank-sum tests were used, as appropriate, to compare participants characteristics across baseline categories and trajectory groups. Tests were conducted at the level of significance α = 5%.

## Results

A total of 1538 women with at least three antenatal BP readings (median number of measurements 5, interquartile range, IQR = 4–8) and a live singleton birth were included in this analysis: 967 HIV-uninfected (63%) and 571 HIV-infected (37%); 54% of the latter group initiated ART pre-conception (*n* = 306) and 46% during pregnancy (*n* = 265) (Table 1). HIV-infected women were older, less likely to be primigravid and more likely to be anaemic than HIV-uninfected women (Table 1). HIV-infected women who initiated ART before pregnancy were older, less likely to be primigravid and anaemic than those who initiated during pregnancy. Overall, 25% of women were overweight and 50% obese, similar by HIV status and timing of ART initiation (Table 1). The median gestational age at the first ANC visit was 18 weeks (IQR 13–24) with no differences by HIV status; 29% of the women were in their first trimester of pregnancy while 59 and 12% were in their second and third trimester respectively.

**Table 1:** Baseline description of women with at least 3 pre-labour blood pressure readings

	Total ***N*** = 1538	HIV- uninfected ***N*** = 967	HIV- infected ***N*** = 571	***P*** value*	HIV-infected ***N*** = 571		***P*** value**
					Initiated before pregnancy ***N*** = 306	Initiated during pregnancy ***N*** = 265	
Age, years (%)				< 0.0001			< 0.0001
< 24	429 (28)	329 (34)	100 (18)		47 (15)	53 (20)	
25–29	454 (30)	290 (30)	164 (29)		71 (23)	93 (35)	
> 30	655 (43)	348 (36)	307 (54)		188 (61)	119 (45)	
Median (IQR)	28 (24–32)	27 (23–31)	30 (26–34)		31 (27–35)	29 (25–32)	
Height, cm (%)				0.177			0.430
≤ 155	470 (30)	301 (31)	263 (30)		87 (28)	82 (31)	
156–161	533 (35)	341 (35)	291 (33)		102 (33)	90 (34)	
≥ 162	412 (27)	242 (25)	250 (30)		93 (30)	77 (29)	
*Missing*	*123 (8)*	*141 (9)*	*53 (7)*		*24 (8)*	*16 (6)*	
Median (IQR)	158 (154–162)	158 (154–162)	158 (154–163)		158 (154–163)	158 (155–163)	
Body Mass Index, kg/m^2^ (%)				0.074			0.121
Underweight (< 18.5)	9 (0.6)	4 (0.4)	5 (0.9)		3 (1)	2 (0.8)	
Normal (18.5–24.9)	246 (16)	144 (15)	102 (18)		49 (16)	53 (20)	
Overweight (25.0–29.9)	386 (25)	241 (25)	145 (25)		85 (28)	60 (23)	
Moderately Obese (30.0–34.9)	364 (24)	221 (23)	143 (25)		80 (26)	63 (24)	
Severely Obese (> 35.0)	397 (26)	270 (28)	127 (22)		59 (19)	68 (26)	
*Missing*	*136 (9)*	*87 (9)*	*49 (9)*		*30 (10)*	*19 (7)*	
Median (IQR)	31 (26–36)	31 (27–37)	30 (26–35)		30 (26–34)	31 (26–36)	
Gravidity (%)				0.001			0.001
1	291 (19)	210 (22)	81 (14)		35 (11)	46 (17)	
2	575 (37)	363 (37)	212 (37)		106 (35)	106 (40)	
≥ 3	666 (43)	393 (41)	273 (48)		162 (53)	111 (42)	
*Missing*	*6 (0.4)*	*1 (0.1)*	*5 (0.9)*		*3 (1)*	*2 (0.8)*	
Median (IQR)	2 (2–3)	2 (2–3)	2 (2–3)		3 (2–3)	2 (2–3)	
Parity (%)				0.004			< 0.0001
0	383 (25)	268 (28)	115 (20)		56 (18)	59 (22)	
1	644 (42)	396 (41)	248 (43)		121 (40)	127 (48)	
≥ 2	505 (33)	302 (31)	203 (36)		126 (41)	77 (29)	
*Missing*	*6 (0.4)*	*1 (0.1)*	*5 (0.9)*		*3 (1)*	*2 (0.8)*	
Median (IQR)	1 (1–2)	1 (0–2)	1 (1–2)		1 (1–2)	1 (1–2)	
Previous Preterm (%)				0.230			0.368
Yes	127 (8)	74 (8)	53 (9)		26 (9)	27 (10)	
Haemoglobin g/dl (%)				< 0.0001			< 0.0001
Normal (≥11.0)	567 (37)	369 (38)	199 (35)		119 (39)	80 (30)	
Mild Anaemia (9–10.9)	401 (26)	238 (25)	163 (29)		81 (26)	82 (31)	
Moderate Anaemia (7–8.9)	94 (6)	42 (4)	52 (9)		19 (6)	33 (12)	
Severe Anaemia (< 7)	2 (0.1)	2 (0.2)	0		0	0	
*Missing*	*474 (32)*	*317 (33)*	*157 (28)*		*87 (28)*	*70 (26)*	
Gestational Age Assessment^a^							
Median (weeks) (IQR)	18 (13–24)	18 (13–24)	18 (12–24)	0.252	17 (12–23)	19 (13–24)	0.334
Booking Blood Pressure (mmHG) (%)				0.967			0.575
Normal (Sys < 120 or Dia < 80)	989 (64)	622 (64)	367 (64)		196 (64)	171 (65)	
Prehypertensive (Sys 120–139 and Dia 80–89)	471 (31)	297 (31)	174 (31)		98 (32)	76 (29)	
Hypertensive (Sys > 140 or Dia > 90)	78 (5)	48 (5)	30 (5)		12 (4)	18 (7)	
Booking Mean arterial pressure (mmHG)				0.527			0.587
Low (< 65)	26 (2)	16 (2)	10 (2)		4 (1)	6 (2)	
Normal (65–99)	1428 (93)	903 (93)	525 (92)		285 (93)	240 (91)	
High (> 100)	84 (5)	48 (5)	36 (6)		17 (6)	19 (7)	
Number of antenatal BP measures	5 (4–8)	5 (4–8)	6 (4–8)	0.008			
Any hypertension	337 (22)	208 (22)	129 (23)	0.620	62 (20)	67 (25)	0.311
Hypertension type^b^ (%)							
Chronic	42 (13)	28 (13)	14 (11)	0.560	4 (7)	10 (15)	0.480
Gestational	284 (84)	172 (83)	112 (87)		56 (90)	56 (84)	
Unknown	11 (3)	8 (4)	3 (2)		2 (3)	1 (1)	
Diagnostic Criteria (%)				0.377			0.417
Both SBP ≥140 mmHg and DBP ≥90 mmHg	40 (12)	26 (13)	14 (11)		6 (10)	8 (12)	
Only SBP ≥140 mmHg	133 (39)	87 (42)	46 (36)		26 (42)	20 (30)	
Only DBP ≥90 mmHg	164 (49)	95 (46)	69 (53)		30 (48)	39 (58)	
Severity (%)				0.570			0.849
Non-severe: SBP 140–159 or DBP 90-109 mmHg	324 (96)	199 (96)	125 (97)		60 (97)	65 (97)	
Severe: BP ≥ 160 or DBP ≥110 mmHg)	253 (4)	9 (4)	4 (3)		2 (3)	2 (3)	

* Comparison between HIV-uninfected vs HIV-infected

** Comparison between HIV-uninfected vs HIV-infected (Initiated before pregnancy) vs HIV-infected (Initiated during pregnancy)

^a^ Best available measure (USS, SFH, LMP)

^b^ Chronic hypertension incidence estimated among women who were assessed at < 20 weeks gestation. Gestational hypertension incidence will be estimated among women previously assessed as normotensive at < 20 weeks or assessed for the first time at 20 weeks

At the first ANC visit, 31% of women were classified as prehypertensive and 5% hypertensive, with no differences by HIV status. Differences were however observed by ART status with a higher proportion of women initiating ART in pregnancy classified as hypertensive compared to those initiating preconception (7% vs 4%). Using the cross-sectional hypertension assessments at multiple time points antenatally, 22% of women were classified as being hypertensive at least once with no differences according to HIV/ART status. For all those who were hypertension at any point, 13% were hypertensive < 20 weeks GA and thus classified as having chronic hypertension; 87% were either initially assessed to be normotensive at < 20 GA and were subsequently hypertensive, or were assessed > 20 weeks GA as hypertensive and classified as having gestational hypertension (Table 1).

### Joint blood pressure trajectory groups

**Fig. 1:**
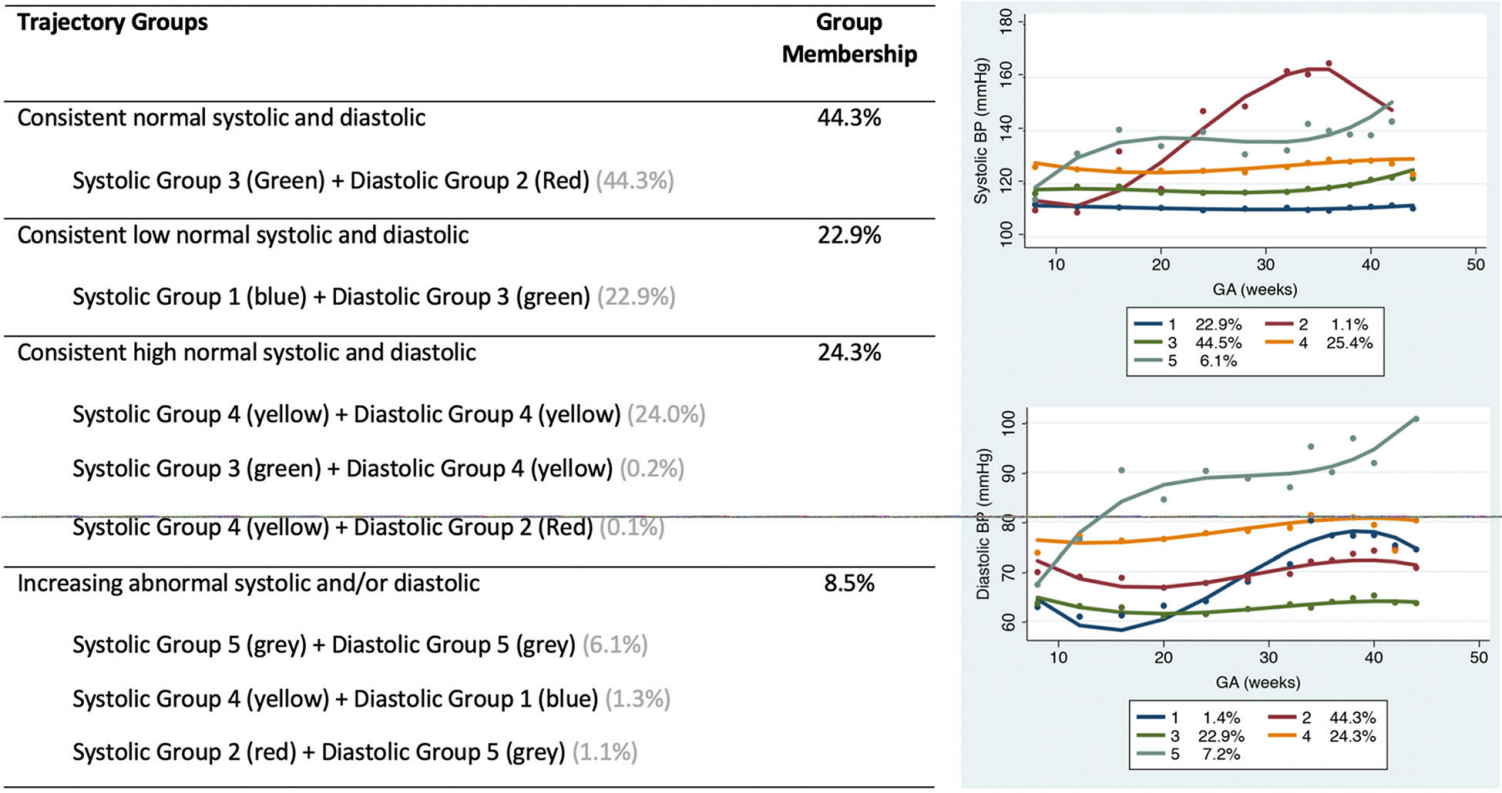
Joint trajectory group combinations: Five systolic (**a**) and five diastolic (**b**) blood pressure joint trajectory group combinations were grouped and classified according to longitudinal trend and mean start BP; into four joint groups: consistent normal systolic and diastolic BP, consistent low normal systolic and diastolic BP, consistent high normal systolic and diastolic BP, and increasing abnormal systolic and/or diastolic BP

Preliminary analysis was conducted using single trajectory models, in order to identify the optimal degree of the polynomials; based on the lowest BIC criterion, the quadratic function fit the data best. For the joint SBP and DBP models using the lowest BIC criterion, the 5-class model was the best fitting (Suppl. Table S1), with model fit statistics indicating this to be an adequately fitting model (Suppl. Table S2). When assessing the probability matrix of membership for the five SBP and five DBP classes combinations, only seven of the possible 25 combinations had at least one individual with that joint trajectory (Suppl. Table S3). Based on clinical input, these seven SBP and DBP combinations were grouped and classified according to the longitudinal trend and the mean start BP. The four joint groups created were: consistent normal (SB*P* values persistently around 110 mmHg, DBP values persistently around 70 mmHg; *n* = 767 (44.3%)), consistent low normal (SBP values persistently around 100 mmHg and DBP values persistently around 60 mmHg; *n* = 384 (23%)), consistent high normal (SBP values either around 110 mmHg or slightly increasing to between 120 and 140 mmHg and DBP values either around 70 mmHg or slightly increasing to between 75 and 80 mmHg; *n* = 316 (24%)), and increasing abnormal (both SBP and DBP values initially at normal levels then increasing (with differing magnitudes) during observation period; *n* = 71 (9%)) BP (Fig. 1). The abnormal trajectory group likely contains women who experience pre-eclampsia, which is characterized by a sudden, substantial increase in BP during the latter parts of pregnancy, along with evidence of end-organ and/or placental dysfunction (which we were unable to assess).

### BP trajectory group predictors

Using the normal trajectory group as reference, HIV-infected women were more likely to be in the low normal trajectory group (aRR 1.24, 95% CI 0.94–1.64) than HIV-uninfected women (Suppl. Table S4). A higher proportion of older women (> 30 years) were in the high normal and abnormal trajectory groups. Body mass index (BMI) status appeared to be associated with trajectory groups: women who were moderately and severely obese were more likely to be in the high normal (27 and 34% respectively) or abnormal (25 and 39% respectively) trajectory groups, compared to normal and overweight women who were more likely to be in the low normal (27 and 31% respectively) group (Suppl. Table S4). In multivariable analysis (adjusted for age, BMI, HIV status and gravidity), with the normal trajectory group as reference, older (aRR 1.52, 1.11–2.10) and obese (aRR 2.06, 1.31–3.25) women were at increased risk of being in the high normal group.

Among HIV-infected women, no differences were observed according to timing of initiation in the normal, low normal and high normal trajectory groups. However, among the women with abnormal trajectories, they were more likely to have initiating ART during pregnancy compared to initiating preconception (20% vs 10%) (Suppl. Table S5).

About a third of women in the high normal (35%) and abnormal trajectory groups (31%) were classified as being normotensive at their first ANC, highlighting the problem with only using cross-sectional BP assessments. A significant proportion of women in the high normal and abnormal trajectory groups were classified as being pre-hypertensive at the first ANC, 53 and 42% respectively (Suppl. Table S5). The majority of women classified as being hypertensive at the first ANC were in the high normal (11%) and abnormal (27%) trajectory groups; with a third of women in abnormal trajectory group also classified has having a high mean arterial pressure.

When hypertension was assessed throughout the antenatal period, the majority of women in the high normal (51%) and abnormal (97%) trajectory group met the conventional hypertensive cut off at least once (Table 2). When grouped according to onset of hypertension most women with hypertension in all trajectory groups were classified as having gestational hypertension.

**Table 2:** Hypertension Description by trajectory group

	Total ***N*** = 1538	Group 1: Normal ***N*** = 767	Group 2: Low Normal ***N*** = 384	Group 3: High Normal ***N*** = 316	Group 4: Abnormal ***N*** = 71	***P*** value
Number of measures	5 (4–8)	5 (4–7)	5 (4–7)	6 (4–10)	7 (4–16)	< 0.0001
Any hypertension (%)	337 (22)	93 (12)	14 (4)	161 (51)	69 (97)	< 0.0001
Hypertension type* (%)						
Chronic	42 (13)	15 (16)	2 (14)	23 (14)	2 (3)	0.001
Gestational	284 (84)	70 (75)	11 (79)	136 (85)	67 (97)	
Unknown	11 (3)	8 (9)	3 (2)	2 (1)	0	
Diagnostic Criteria (%)						< 0.0001
Both SBP ≥140 mmHg and DBP ≥90 mmHg	40 (12)	5 (5)	0	12 (7)	23 (33)	
Only SBP ≥140 mmHg	133 (39)	46 (50)	7 (50)	66 (41)	14 (20)	
Only DBP ≥90 mmHg	164 (49)	42 (45)	7 (50)	83 (52)	32 (47)	
Severity (%)						0.578
Non-severe (SBP 140–159 or DBP 90-109 mmHg)	324 (96)	89 (96)	13 (93)	157 (98)	65 (94)	
Severe (SBP ≥ 160 or DBP ≥110 mmHg)	253 (4)	4 (4)	1 (7)	4 (2)	4 (6)	

* Chronic hypertension incidence estimated among women who were assessed at < 20 weeks gestation. Gestational hypertension incidence will be estimated among women previously assessed as normotensive at < 20 weeks. Unknown = assessed for the first time at 20 weeks

### BP trajectory groups and adverse birth outcomes

Comparing outcomes overall between the joint trajectory groups, a higher incidence of any PTD (unadjusted, uRR3.13, 95% CI 2.35–4.17; 50%) and any LBW (uRR 2.81, 95% CI 1.90–4.15; 32%) was observed among women in the abnormal trajectory group (Table 3). The lowest risk of PTD was observed in the consistent low normal group (uRR 0.67, 95% CI 0.48–0.94; 11%). There was a fairly even split between late and moderately preterm among the normal (37% vs 43%) and low normal (46% vs 34%); trajectory groups, but in contrast there was a higher incidence of moderate than late preterm in the high normal (53% vs 25%) and abnormal (46% vs 26%) trajectory groups. Further, 29% of the preterm deliveries in the abnormal trajectory group were classified as very preterm. Among women with PTD, there was no appreciable difference in delivery mode of women in the normal and high normal trajectory groups; however in contrast in the low normal and abnormal trajectory groups PTD was commonly associated with emergency caesarean section, indicating a suspicion of adverse pregnancy outcome necessitating preterm delivery.

**Table 3:** Birth Outcomes by joint trajectory groups

	Total	Group 1: Normal	Group 2: Low Normal	Group 3: High Normal	Group 4: Abnormal	***P*** value*
Gestational Age (weeks) (%)	*N* = 1532	*N* = 764	*N* = 382	*N* = 316	*N* = 70	< 0.0001
Term (≥ 37)	1262 (82)	642 (84)	341 (89)	244 (77)	35 (50)	
Any Preterm (< 37)	270 (18)	122 (16)	41 (11)	72 (23)	35 (50)	
Late Preterm (34–37)	93 (6)	45 (6)	19 (5)	20 (7)	9 (13)	
Moderately Preterm (32–34)	120 (8)	52 (7)	14 (4)	38 (12)	16 (23)	
Very Preterm (28–32)	57 (4)	25 (3)	8 (2)	14 (4)	10 (14)	
Birthweight (g) (%)	*N* = 1530	*N* = 763	*N* = 384	*N* = 312	*N* = 71	< 0.0001
Normal (≥2500)	1333 (87)	675 (88)	342 (89)	268 (86)	48 (68)	
Any LBW (< 2500)	197 (13)	88 (12)	42 (11)	44 (14)	23 (32)	
LBW (< 2500)	159 (10)	75 (10)	37 (10)	34 (11)	13 (18)	
Very LBW (< 1500)	38 (3)	13 (2)	5 (1)	10 (3)	10 (14)	
Mean (SD)	3116 (644)	3146 (616)	3158 (550)	3110 (686)	2580 (934)	

All the variables had < 4% missing data, with similar proportions of missing data across the comparison groups

In the normal, low normal and high normal trajectory groups the incidence of LBW was similar (12% vs 11% vs 14%), whereas a significantly higher incidence was observed in the abnormal trajectory group (32%). In the normal, low normal and high normal trajectory groups, of the LBW neonates, 12 and 23% were VLBW, whereas in the abnormal trajectory group 44% were VLBW (Table 2). In multivariable analyses allowing for other variables potentially associated with PTD or LBW, with the normal group as reference, a low normal trajectory (aRR0.59, 0.41–0.85) was associated with decreased risk of PTD, high normal (aRR1.48, 1.12–1.95) and abnormal trajectories with increased risk of PTD (aRR3.18, 2.32–4.37). The abnormal trajectory was also significantly associated with increased LBW risk (aRR1.78, 1.22–2.59) (Table 4).

**Table 4:** Adjusted Associations between joint trajectory groups and adverse birth outcomes

Trajectory Group (Ref cat: Normal)	Preterm^**a**^			Low Birthweight^b^		
	RR (95% CI)	ARR (95% CI)	***P***-value	RR (95% CI)	ARR (95% CI)	***P***-value
Low Normal	0.67 (0.48–0.94)	0.59 (0.41–0.85)	0.004	0.95 (0.67–1.34)	0.99 (0.70–1.43)	0.998
High Normal	1.43 (1.10–1.85)	1.48 (1.12–1.95)	0.005	1.22 (0.87–1.71)	1.16 (0.83–1.61)	0.390
Abnormal	3.13 (2.35–4.17)	3.18 (2.32–4.37)	< 0.0001	2.8**1** (1.90–4.15)	1.78 (1.22–2.59)	0.003

^a^adjusted for age, body mass index and previous preterm delivery

^b^adjusted for age, body mass index and gestational age at delivery

In stratified analyses to explore potential effect modification by HIV status, with the normal group as reference, among HIV-uninfected women low normal trajectory was associated with significantly decreased risk of PTD (aRR0.48, 0.29–0.81) while high normal (aRR1.53, 1.08–2.16) and abnormal trajectories (aRR3.42, 2.31–5.05) were associated with significantly increased risk of PTD. Among HIV-infected women these associations were similar but did not reach statistical significance (low normal and PTD: aRR 0.74, 95% CI 0.44–1.26; high normal and PTD: aRR1.40 95% CI 0.90–2.19; abnormal and PTD: aRR2.66 95% CI 1.49–4.76) (Table 5). Surprisingly, HIV-infected women in the high normal and abnormal trajectory groups had a lower risk for PTD compared to HIV-uninfected women, the limited sample size of HIV-infected women could have contributed to the imprecision of the stratified estimates.

**Table 5:** Adjusted Associations between joint trajectory groups and adverse birth outcomes stratified by HIV status

Trajectory Group (Ref cat: Normal)	Preterm^**a**^			Low Birthweight^**b**^		
	HIV-uninfected ARR (95% CI)^**†**^	HIV-infected ARR (95% CI)^**††**^	***P***-value	HIV-uninfected ARR (95% CI)^**†**^	HIV-infected ARR (95% CI)^**††**^	***P***-value
Low Normal	0.48 (0.29–0.81)	0.74 (0.44–1.26)	0.005^**†**^ 0.267^**††**^	0.75 (0.46–1.22)	1.37 (0.80–2.33)	0.244^**†**^ 0.252^**††**^
High Normal	1.53 (1.08–2.16)	1.40 (0.90–2.19)	0.017^**†**^ 0.134^**††**^	1.13 (0.75–1.70)	1.18 (0.70–2.01)	0.549^**†**^ 0.531^**††**^
Abnormal	3.42 (2.31–5.05)	2.66 (1.49–4.76)	< 0.0001† 0.001^**††**^	2.01 (1.33–3.02)	1.08 (0.43–2.72)	0.001^**†**^ 0.872^**††**^

^a^adjusted for age, body mass index and previous preterm delivery

^b^adjusted for age, body mass index and gestational age at delivery

^†^p-value for the association between adverse birth outcome and trajectory group categories among HIV-uninfected women

^††^p-value for the association between adverse birth outcome and trajectory group categories among HIV-infected women

There appeared to be evidence of effect measure modification on the relative risk scale by HIV status in the relationship between the abnormal trajectory group and LBW infants: among HIV-uninfected women in the abnormal trajectory group there was an increased risk of LBW (aRR2.01, 95% CI 1.33–3.02), while in the HIV-infected women this effect was attenuated and did not reach statistical significance (aRR1.08, 95% CI 0.43–2.72) (Table 5).

## Discussion

In this prospective cohort of HIV-infected and –uninfected pregnant women at a large public sector primary care antenatal facility in South Africa, we identified groups with distinct antenatal BP trajectories, which were not associated with HIV status; both high consistent and increasing abnormal BP trajectories were associated with subsequent risk of PTD and LBW. Understanding different BP trajectories in pregnancy, especially in areas with high HIV prevalence, which has been shown to be associated with BP, will inform efforts to identify women at increased risk of developing HDP and adverse birth outcomes.

Research studies tend to use stringent criteria (traditional cut-offs), with a strong emphasis on correctly ruling out those without hypertension from being classified as hypertensive. Additionally, these studies usually predict subsequent HDP development based on BP measured at a single time point such at the first ANC visit [24, 25]. Using such a cross-sectional approach, 5% of women in our cohort met the hypertension cut-off at their first ANC visit while 22% were classified as hypertensive at any point in pregnancy; substantially higher than levels observed in a trial conducted in southern Asia and sub-Saharan Africa [26]. In contrast, clinically all-encompassing and practical criteria are predominantly used to classify HDP, even if they have higher false positivity rates, because the ultimate goal is to identify women with a higher than average risk to themselves or their fetuses in order to guide clinical management [27]. This approach is more informative as it differentiates between new onset HDP, which can affect maternal and infant morbidity and mortality [28], and isolated new-onset SBP and/or DBP elevation, which is quite common during pregnancy [29].

The GBTM approach is well-suited to explore trajectories for outcomes that are commonly categorised, such the natural history of BP in pregnancy. Using this approach, we identified distinct trajectory groups which we classified according to the mean BP values relative to clinical definitions of BP levels and the pattern of change throughout the antenatal period (consistent or increasing). The patterns observed in the low normal, normal and high normal trajectory groups resembles patterns seen in clinically healthy women, where BP decreases steadily until mid-pregnancy then rises again returning to preconception levels in late pregnancy until delivery [13]. In contrast, clinical reports show that in women who develop gestational hypertension, BP remains stable until mid-pregnancy then increases until delivery [13, 30] – the abnormal trajectory group resembles this pattern. This group appeared to be a combination of women with chronic hypertension (likely classified as hypertensive at baseline (27%)), and women with gestational hypertension (42% prehypertensive, 31% normotensive at baseline). These findings suggest that similar to the approach applied clinically, a better approach for risk assessment in research studies would be incorporation of BP measurements throughout pregnancy to assess trajectory of BP before delivery rather than the traditional cross-sectional measures.

We found that BP characteristics were similar in HIV-infected and HIV-uninfected women, half of whom were on ART from before pregnancy and half from ~ 16 weeks of pregnancy, with no differences in the incidence of hypertension at baseline or throughout pregnancy. Studies investigating the association between HIV and HDP have shown conflicting results with either increased risk [6] or no difference in risk [31, 32]. Though limited, data on specific ART drug classes has shown exposure to PIs to be strongly associated with hypertension [6, 33]; in our setting nucleoside reverse transcriptase inhibitors regimens are predominantly used rather than PIs which could explain lack of difference by HIV status. Our findings by HIV status are similar to a retrospective cohort study of two overlapping pregnancy cohorts [34], but differed according to ART status. We found that women classified as hypertensive or as having abnormal trajectories were more likely to have initiated ART in pregnancy; and this could possibly be linked to immune reconstitution, a hyper-inflammatory state associated with ART initiation [35, 36], vascular dysfunction and alterations in sympathetic nervous outflow [37]. This process is exacerbated by the presence of well-known risk factors including extreme reproductive ages, obesity and genetic predisposition. In our cohort, we confirm increased maternal age and BMI to be associated with hypertension, consistent with other studies [38, 39]. Additionally, women who were overweight or obese were more like to have a high normal BP trajectory.

High blood pressure in pregnancy is associated with adverse pregnancy outcomes for both mother and infant. Similar to findings from Ethiopia, we found that approximately a third of women classified as hypertensive at 1st ANC visit delivered preterm and LBW infants [40].

However, given that only 5% of women were classified as hypertensive at first ANC (using traditional cut-offs) this cross-sectional assessment approach may not correctly identify women at increased adverse birth outcome risk. In contrast, using GBTM enabled identification of women who were not classified as hypertensive but who were at increased risk, as such the HDP and adverse birth outcomes association is likely to be more accurate using this approach.

We found that women with high normal and abnormal trajectories were at increased risk of PTD; while those with abnormal trajectories were at increased LBW risk. This is consistent with previous data showing that among women without pre-existing hypertension or preeclampsia, higher maternal blood pressure levels were associated with impaired fetal growth from the third trimester onward as well as increased risks of adverse birth outcomes [30, 41]. Increased PTD incidence among women with abnormal BP levels is expected because of pre-emptive clinical management protocols that prioritise maternal safety, and thus may necessitate preterm delivery to prevent severe complications of HDP, which could explain the association between PTD and emergency caesarean sections in our cohort. The association between LBW and HDP has been postulated to be attributable to both the effect of early delivery and/or fetal growth restriction [42]. The mechanisms by which maternal blood pressure levels can affect fetal growth are unclear, with suggestions that it could be related to placental dysfunction or adverse maternal cardiovascular adaptations to pregnancy [30]. Separately, associations between HIV/ART and BP and adverse birth outcomes have been observed, however in our study where we assessed HIV status as an effect modifier of the relationship between BP trajectories and adverse birth outcomes, we observed no differences by HIV status. While associations were attenuated for HIV-infected women, we hypothesise that limited sample size rather than an HIV effect are the explanation - an assumption further substantiated by the lack of BP differences by HIV status.

This analysis is among the first to explore discrete latent class assignment of BP trajectory groups during pregnancy and their relationship with adverse birth outcomes, in a population-based study. Strengths of the study include the use of prospectively collected blood pressure and gestational age measurements at baseline and throughout pregnancy, which enabled the distinction between women with chronic and gestational hypertension based on gestation-dependent BP changes. Additionally, GBTM is an advantageous analytic approach because it does not rely on predefined categorisation of trajectories, removing subjectivity regarding class assignment, and enabling capturing of within-class heterogeneity that might be missed with analyses based on class-averaged means [22]. It should however be noted that the trajectory grouping using this approach depends on the data, and while the trajectory groups may provide a general trend of BP control over time, they may not accurately characterise each individual’s actual BP trajectory. Limitations include the availability of only basic maternal characteristics for use in adjusted analyses with no data on other pre-pregnancy risk factors for HDP such as hypertension family history, previous HDP in multipara women; and no proteinuria data to establish whether the women in abnormal trajectory group had preeclampsia. Lacking detailed preconception and early pregnancy maternal data, we were unable to find significant association with baseline characteristics and trajectory, except for maternal BMI and gravidity. Data on factors possibly associated with birth outcome such as gestational diabetes could not be taken into account because they were not available in complete and systematic manner. Additionally, blood pressure measurements were collected during routine clinical practice, where the use of a single BP measure may have greater measurement error than standardised measures. This analysis provides some of the first insights into the relationships between BP trajectory levels and their association with adverse birth outcomes, in particular the use of trajectories enables identification of women at increased risk of adverse birth outcomes who would not be picked up using the traditional approach currently employed in studies investigating this association.

## Conclusions

Globally, HDP incidence has increased over time and as obesity increases in low- and middle-income countries, high blood pressure during pregnancy has the potential to adversely impact maternal and child health outcomes. It is important for public health to monitor trends in pregnancy outcomes by HDP. We detected heterogeneity in general relationship between HDP and PTD/LBW outcomes, by identifying five groups with distinctive patterns of BP trends over the pregnancy. Further methodology work is required with larger datasets in order establish where precisely divergence between trajectories occurs in order to inform optimum timing of BP measurements in studies investigating HDP. Larger datasets would also enable identification of heterogeneity within trajectory groups particularly among the high normal and abnormal trajectories. Further work is also required to inform understanding of different BP trajectories in pregnancy, particularly in high HIV prevalent settings were ART use is linked to weight gain which may extenuate BP levels, in order to provide appropriate surveillance and counselling to HIV-infected pregnant women and those with pregnancy intentions.

## Supplementary Information

**Additional file 1 Supplementary Table S1** Average posterior probabilities of joint trajectory class assignments. **Supplementary Table S2** Model fit statistics of joint trajectory classes. **Supplementary Table S3** Joint systolic and diastolic blood pressure trajectory group membership combinations. **Supplementary Table S4** Adjusted associations between baseline predictors and joint trajectory groups. **Supplementary Table S5** Baseline descriptions of women with at least 3 pre-labour blood pressure readings according to trajectory group

## Data Availability

The data collected will be available to external investigators upon submission and approval of a data analysis plan. Requests for data available within PIMS should be submitted to M.Newell@soton.ac.uk and Landon.Myer@uct.ac.za and will be reviewed by the study steering committee.

## Abbreviations

BP: Blood Pressure
HDP: Hypertensive Disorders in pregnancy
SBP: Systolic Blood Pressure
DBP: Diastolic Blood Pressure
MAP: Mean arterial pressure
ART: Antiretroviral Therapy
PI: Protease Inhibitors
ANC: Antenatal Care
GA: Gestational Age
LMP: Last Menstrual Period
SFH: Symphysis-Fundal Height
PTD: Preterm Delivery
LBW: Low Birthweight
VLBW: Very Low Birthweight
GBTM: Group-based Trajectory Modelling
BIC: Bayesian Information Criterion
BMI: Body Mass Index
