# Supplementary material for: Blood pressure trajectories during pregnancy and associations with adverse birth outcomes among HIV-infected and HIV-uninfected women in South Africa: a group-based trajectory modelling approach
Source: BMC Pregnancy Childbirth. 2020 Nov 30;20:742. doi: 10.1186/s12884-020-03411-y (PMC7708197; doi:10.1186/s12884-020-03411-y)
Supplement: Supplementary file 1 — Additional file 1 Supplementary Table S1 Average posterior probabilities of joint trajectory class assignments. Supplementary Table S2 Model fit statistics of joint trajectory classes. Supplementary Table S3 Joint systolic and diastolic blood pressure trajectory group membership combinations. Supplementary Table S4 Adjusted associations between baseline predictors and joint trajectory groups. Supplementary Table S5 Baseline descriptions of women with at least 3 pre-labour blood pressure readings according to trajectory group [file 12884_2020_3411_MOESM1_ESM.docx]

Supplementary Table 1: Average posterior probabilities of joint trajectory class assignments

| Classes | Model 1 | | | | Model 2 | | | |
| --- | --- | --- | --- | --- | --- | --- | --- | --- |
|  | Number of subjects (%) | Average Posterior Probability (%) |  | Number of Subjects (%) | | Average Posterior Probability (%) |  |  |
| 1 | 1118 (73) | 94.23 | 0.998 (0.953-0.999) | 1122 (73) | | 94.38 | 0.998 (0.954-0.999) |  |
| 2 | 420 (27) | 89.86 | 0.977 (0.842-0.999) | 416 (27) | | 89.59 | 0.977 (0.840-0.999) |  |

| Classes | Model 1 | | | Model 2 | | |
| --- | --- | --- | --- | --- | --- | --- |
|  | Number of subjects (%) | Average Posterior Probability (%) |  | Number of Subjects (%) | Average Posterior Probability (%) |  |
| 1 | 611 (40) | 85.58 | 0.919 (0.741-0.988) | 810 (53) | 90.10 | 0.981 (0.863-0.999) |
| 2 | 810 (52) | 90.10 | 0.981 (0.863-0.999) | 611 (40) | 85.80 | 0.921 (0.745-0.989) |
| 3 | 117 (8) | 91.26 | 0.981 (0.875-0.999) | 117 (7) | 97.45 | 0.975 (0.846-0.999) |

| Classes | Model 1 | | | | Model 2 | | | | | | |  |  |
| --- | --- | --- | --- | --- | --- | --- | --- | --- | --- | --- | --- | --- | --- |
|  | Number of subjects (%) | Average Posterior Probability (%) | |  | | | Number of Subjects (%) | | Average Posterior Probability (%) | |  | | |
| 1 | 391 (26) | 85.51 | 0.914 (0.749-0.991) | | | 775 (50) | | 81.83 | | 0.866 (0.722-0.951) | | |  |
| 2 | 775 (50) | 82.24 | 0.868 (0.725-0.952) | | | 391 (26) | | 85.51 | | 0.913 (0.749-0.991) | | |  |
| 3 | 310 (20) | 86.62 | 0.866 (0.703-0.981) | | | 310 (20) | | 83.22 | | 0.874 (0.705-0.983) | | |  |
| 4 | 62 (4) | 89.46 | 0.987 (0.832-0.999) | | | 62 (4) | | 89.46 | | 0.988 (0.832-0.999) | | |  |

| Classes | Model 1 | | | | Model 2 | | | |
| --- | --- | --- | --- | --- | --- | --- | --- | --- |
|  | Number of subjects (%) | Average Posterior Probability (%) |  | Number of Subjects (%) | | Average Posterior Probability (%) |  |  |
| 1 | 384 (25) | 85.24 | 0.914 (0.749-0.990) | 9 (0.2) | | 99.80 | 0.999 (0.999-1.000) |  |
| 2 | 9 (1) | 94.48 | 0.973 (0.947-0.999) | 768 (50) | | 81.53 | 0.863 (0.716-0.948) |  |
| 3 | 768 (50) | 81.80 | 0.865 (0.722-0.950) | 384 (25) | | 85.24 | 0.914 (0.749-0.990) |  |
| 4 | 318 (20) | 83.32 | 0.866 (0.714-0.976) | 318 (20) | | 80.49 | 0.852 (0.671-0.960) |  |
| 5 | 59 (4) | 82.39 | 0.890 (0.672-0.974) | 59 (4) | | 85.93 | 0.938 (0.683-0.997) |  |

Supplementary Table 2: Model fit statistics of joint trajectory classes

| Model | BIC (N=73074) | BIC (n=10276) | AIC | >5% per group |
| --- | --- | --- | --- | --- |
| 2 Classes | -97870.52 | -97849.93 | -97773.93 | Yes |
| 3 Classes | -94728.19 | -94694.84 | -94571.80 | Yes |
| 4 Classes | -93533.43 | -93485.36 | -93308.04 | Yes |
| 5 Classes | -93135.40 | -93070.67 | -92831.83 | No |

Supplementary Table 3: Joint Systolic and Diastolic blood pressure trajectory group membership combinations

| Joint group membership combinations* | | | Prevalence | Combination Present |
| --- | --- | --- | --- | --- |
|  | Model 1  (Systolic BP) | Model 2 (Diastolic BP) |  |  |
| 1 | 1 | 1 | 0.0% | No |
| 2 | 2 | 1 | 0.0% | No |
| 3 | 3 | 1 | 0.0% | No |
| 4 | **4** | **1** | **1.4%** | **Yes** |
| 5 | 5 | 1 | 0.0% | No |
| 6 | 1 | 2 | 0.0% | No |
| 7 | 2 | 2 | 0.0% | No |
| 8 | **3** | **2** | **44.3%** | **Yes** |
| 9 | 4 | 2 | 0.0% | No |
| 10 | 5 | 2 | 0.0% | No |
| 11 | **1** | **3** | **22.9%** | **Yes** |
| 12 | 2 | 3 | 0.0% | No |
| 13 | 3 | 3 | 0.0% | No |
| 14 | 4 | 3 | 0.0% | No |
| 15 | 5 | 3 | 0.0% | No |
| 16 | 1 | 4 | 0.0% | No |
| 17 | 2 | 4 | 0.0% | No |
| 18 | **3** | **4** | **0.2%** | **Yes** |
| 19 | **4** | **4** | **24.1%** | **Yes** |
| 20 | 5 | 4 | 0.0% | No |
| 21 | 1 | 5 | 0.0% | No |
| 22 | **2** | **5** | **1.1%** | **Yes** |
| 23 | 3 | 5 | 0.0% | No |
| 24 | 4 | 5 | 0.0% | No |
| 25 | **5** | **5** | **6.1%** | **Yes** |

*trajectory groups for both outcomes (systolic and diastolic BP)

Supplementary Table 4: Adjusted Associations between baseline predictors and joint trajectory groups

| **Trajectory Group**  **(Ref cat: Normal)** | **Predictor** | | **ARR (95% CI)** | **P-value** |
| --- | --- | --- | --- | --- |
| Low Normal | HIV positive (Ref cat: HIV negative) | | 1.24 (0.94 - 1.64) | 0.123 |
|  | Age (Ref cat: <24 years) | |  |  |
|  |  | 25-29 | 0.83 (0.57 - 1.12) | 0.320 |
|  |  | >30 | 0.79 (0.53 - 1.17) | 0.240 |
|  | Body Mass Index (Ref cat: normal) | |  |  |
|  |  | Overweight | 0.65 (0.45 - 0.92) | 0.016 |
|  |  | Obese | 0.32 (0.22 - 0.45) | <0.0001 |
|  | Gravidity (Ref cat: 1) | |  |  |
|  |  | 2 | 1.87 (1.24 - 2.83) | 0.003 |
|  |  | >3 | 2.14 (1.35 - 3.39) | 0.001 |
| High Normal | HIV positive (Ref cat: HIV negative) | | 1.16 (0.87 - 1.56) | 0.317 |
|  | Age (Ref cat: <24 years) | |  |  |
|  |  | 25-29 | 0.71 (0.47 - 1.09) | 0.121 |
|  |  | >30 | 1.45 (0.95 - 2.22) | 0.087 |
|  | Body Mass Index (Ref cat: normal) | |  |  |
|  |  | Overweight | 1.44 (0.87 - 2.40) | 0.159 |
|  |  | Obese | 2.14 (1.34 - 3.40) | 0.001 |
|  | Gravidity (Ref cat: 1) | |  |  |
|  |  | 2 | 0.69 (0.46 - 1.06) | 0.091 |
|  |  | >3 | 0.65 (0.41 - 1.03) | 0.068 |
| Abnormal | HIV positive (Ref cat: HIV negative) | | 0.96 (0.54 - 1.68) | 0.875 |
|  | Age (Ref cat: <24 years) | |  |  |
|  |  | 25-29 | 1.28 (0.60 - 2.75) | 0.523 |
|  |  | >30 | 1.77 (0.77 - 4.04) | 0.177 |
|  | Body Mass Index (Ref cat: normal) | |  |  |
|  |  | Overweight | 0.79 (0.29 - 2.20) | 0.656 |
|  |  | Obese | 2.10 (0.91 - 4.89) | 0.084 |
|  | Gravidity (Ref cat: 1) | |  |  |
|  |  | 2 | 0.50 (0.24 - 1.05) | 0.065 |
|  |  | >3 | 0.35 (0.15 - 0.81) | 0.014 |

Supplementary Table 5: Baseline description of women with at least 3 pre-labour blood pressure readings according to trajectory group

|  |  | **Total**  **N=1538** | **Group 1: Normal**  **N=767** | **Group 2:**  **Low Normal**  **N=384** | **Group 3:**  **High Normal**  **N=316** | **Group 4:**  **Abnormal**  **N=71** | **P-value** |
| --- | --- | --- | --- | --- | --- | --- | --- |
| Age, years (%) | |  |  |  |  |  | 0.002 |
|  | <24 | 429 (28) | 219 (29) | 113 (29) | 80 (25) | 17 (24) |  |
|  | 25-29 | 454 (30) | 246 (32) | 119 (31) | 69 (22) | 20 (28) |  |
|  | >30 | 655 (43) | 302 (39) | 152 (40) | 167 (53) | 34 (48) |  |
|  | Median (IQR) | 28 (24-32) | 28 (24-32) | 28 (23-32) | 30 (24-34) | 29 (25-34) |  |
| Height, cm (%) | |  |  |  |  |  | 0.023 |
|  | ≤155 | 470 (30) | 222 (29) | 139 (36) | 85 (27) | 24 (34) |  |
|  | 156-161 | 533 (35) | 277 (36) | 134 (35) | 102 (32) | 20 (28) |  |
|  | ≥162 | 412 (27) | 210 (27) | 83 (22) | 100 (32) | 19 (27) |  |
|  | *Missing* | *123 (8)* | *58 (8)* | *28 (7)* | *29 (9)* | *8 (11)* |  |
|  | Median (IQR) | 158 (154-162) | 158 (154-162) | 157 (153-161) | 159 (155-163) | 157 (153-163) |  |
| Body Mass Index, kg/m^2^ (%) | |  |  |  |  |  | <0.0001 |
|  | Underweight (<18.5) | 9 (0.6) | 4 (0.5) | 3 (0.8) | 2 (0.6) | 0 |  |
|  | Normal (18.5-24.9) | 246 (16) | 110 (14) | 104 (27) | 25 (8) | 7 (10) |  |
|  | Overweight (25.0-29.9) | 386 (25) | 192 (25) | 120 (31) | 65 (21) | 9 (13) |  |
|  | Moderately Obese (30.0-34.9) | 364 (24) | 177 (23) | 83 (22) | 86 (27) | 18 (25) |  |
|  | Severely Obese (>35.0) | 397 (26) | 218 (28) | 42 (11) | 109 (34) | 28 (39) |  |
|  | *Missing* | *136 (9)* | *66 (9)* | *32 (8)* | *29 (9)* | *9 (13)* |  |
|  | Median (IQR) | 31 (26-36) | 31 (27-37) | 28 (24-32) | 33 (29-38) | 34 (30-40) |  |
| Gravidity (%) | |  |  |  |  |  | 0.019 |
|  | 1 | 291 (19) | 151 (20) | 53 (14) | 67 (21) | 20 (28) |  |
|  | 2 | 575 (37) | 293 (38) | 157 (41) | 102 (32) | 23 (32) |  |
|  | ≥3 | 666 (43) | 320 (42) | 173 (45) | 145 (46) | 28 (39) |  |
|  | *Missing* | *6 (0.4)* | *1 (0.1)* | *5 (0.9)* | *2 (0.6)* | *0* |  |
|  | Median (IQR) | 2 (2-3) | 2 (2-3) | 2 (2-3) | 2 (2-3) | 2 (1-3) |  |
| Parity (%) | |  |  |  |  |  | 0.028 |
|  | 0 | 383 (25) | 199 (26) | 75 (20) | 85 (27) | 24 (34) |  |
|  | 1 | 644 (42) | 330 (43) | 173 (45) | 116 (37) | 25 (35) |  |
|  | ≥2 | 505 (33) | 235 (31) | 135 (35) | 113 (36) | 22 (31) |  |
|  | *Missing* | *6 (0.4)* | *1 (0.1)* | *5 (0.9)* | *2 (0.6)* | *0* |  |
|  | Median (IQR) | 1 (1-2) | 1 (0-2) | 1 (1-2) | 1 (0-2) | 1 (0-2) |  |
| Previous Preterm (%) | |  |  |  |  |  | 0.030 |
|  | Yes | 127 (8) | 51 (7) | 31 (8) | 35 (11) | 10 (14) |  |
| Haemoglobin g/dl (%) | |  |  |  |  |  | 0.105 |
|  | Normal (≥11.0) | 567 (37) | 297 (39) | 126 (33) | 116 (37) | 28 (39) |  |
|  | Mild Anaemia (9-10.9) | 401 (26) | 196 (26) | 112 (29) | 76 (24) | 17 (24) |  |
|  | Moderate Anaemia (7-8.9) | 94 (6) | 39 (5) | 36 (9) | 15 (5) | 4 (6) |  |
|  | Severe Anaemia (<7) | 2 (0.1) | 2 (0.3) | 0 | 0 | 0 |  |
|  | *Missing* | *474 (32)* | *233 (30)* | *110 (29)* | *109 (34)* | *22 (31)* |  |
| HIV/ART status (%) | |  |  |  |  |  | 0.204 |
|  | HIV negative | 967 (63) | 494 (64) | 226 (59) | 197 (62) | 50 (70) |  |
|  | HIV positive, preconception ART | 306 (20) | 149 (20) | 83 (22) | 67 (21) | 7 (10) |  |
|  | HIV positive, initiating ART | 265 (17) | 124 (16) | 75 (19) | 52 (17) | 14 (20) |  |
| Gestational Age Assessment* | |  |  |  |  |  | 0.0344 |
|  | Median (weeks) (IQR) | 18 (13-24) | 18 (13-23) | 18 (12-24) | 20 (13-25) | 17 (11-22) |  |
| Booking Blood Pressure (mmHG) (%) | |  |  |  |  |  | <0.0001 |
|  | Normal  (Sys <120 and Dia <80) | 989 (64) | 517 (67) | 338 (88) | 112 (35) | 22 (31) |  |
|  | Prehypertensive  (Sys 120-139 and Dia 80-89) | 471 (31) | 228 (30) | 44 (11) | 169 (53) | 30 (42) |  |
|  | Hypertensive  (Sys >140 or Dia >90) | 78 (5) | 22 (3) | 2 (0.5) | 35 (11) | 19 (27) |  |
| Booking Mean arterial pressure (mmHG) (%) | |  |  |  |  |  | <0.0001 |
|  | Low (<65) | 26 (2) | 10 (1) | 15 (4) | 1 (0.3) | 0 |  |
|  | Normal (65-99) | 1428 (93) | 732 (95) | 369 (96) | 279 (88) | 48 (68) |  |
|  | High (>100) | 84 (5) | 48 (3) | 0 | 36 (11) | 23 (32) |  |

* Best available measure (USS, SFH, LMP)
